# Elevation of autoantibody level against PDCD11 in patients with transient ischemic attack

**DOI:** 10.18632/oncotarget.23653

**Published:** 2017-12-24

**Authors:** Yoichi Yoshida, Hao Wang, Takaki Hiwasa, Toshio Machida, Eiichi Kobayashi, Seiichiro Mine, Go Tomiyoshi, Rika Nakamura, Natsuko Shinmen, Hideyuki Kuroda, Hirotaka Takizawa, Koichi Kashiwado, Ikuo Kamitsukasa, Hideo Shin, Takeshi Wada, Akiyo Aotsuka, Eiichiro Nishi, Mikiko Ohno, Minoru Takemoto, Koutaro Yokote, Sho Takahashi, Jun Matsushima, Xiao-Meng Zhang, Masaki Takiguchi, Yasuo Iwadate

**Affiliations:** ^1^ Department of Neurological Surgery, Graduate School of Medicine, Chiba University, Chiba, Japan; ^2^ Department of Biochemistry and Genetics, Graduate School of Medicine, Chiba University, Chiba, Japan; ^3^ Comprehensive Stroke Center, Chiba University Hospital, Chiba, Japan; ^4^ Department of Anesthesia, The First Affiliated Hospital, Jinan University, Guangzhou, P. R. China; ^5^ Department of Neurosurgery, Chiba Cerebral and Cardiovascular Center, Ichihara, Chiba, Japan; ^6^ Department of Neurosurgery, Sawara Prefectural Hospital, Chiba, Japan; ^7^ Medical Project Division, Research Development Center, Fujikura Kasei Co., Saitama, Japan; ^8^ Port Square Kashiwado Clinic, Kashiwado Memorial Foundation, Chiba, Japan; ^9^ Department of Neurology, Kashiwado Hospital, Chiba, Japan; ^10^ Department of Neurology, Chiba Rosai Hospital, Chiba, Japan; ^11^ Department of Neurology, Chibaken Saiseikai Narashino Hospital, Chiba, Japan; ^12^ Department of Neurosurgery, Higashi Funabashi Hospital, Chiba, Japan; ^13^ Department of Internal Medicine, Chiba Aoba Municipal Hospital, Chiba, Japan; ^14^ Department of Pharmacology, Shiga University of Medical Science, Shiga, Japan; ^15^ Department of Clinical Cell Biology and Medicine, Graduate School of Medicine, Chiba University, Chiba, Japan; ^16^ Clinical Research Center, Chiba University Hospital, Chiba, Japan; ^17^ Department of Diagnostic Pathology, Graduate School of Medicine, Chiba University, Chiba, Japan

**Keywords:** TIA, PDCD11, cerebral infarction, autoantibody, biomarker, Gerotarget

## Abstract

**Background:**

Disease specific autoantibodies have been detected in the sera of patients with atherosclerosis-related diseases, such as cerebral infarction, cardiovascular disease. In the present study, we aimed to identify novel autoantibodies responsible for transient ischemic attack (TIA), a prodromal condition for cerebral infarction.

**Methods:**

To identify candidate antigens, we screened a human aortic endothelial cell cDNA library using sera from 20 patients with TIA. Serum antibody levels were measured using amplified luminescent proximity homogeneous assay-linked immunosorbent assay (AlphaLISA) in 2 independent patient/healthy donor (HD) cohorts (*n* = 192 and *n* = 906 in the second screening and validation cohort, respectively).

**Results:**

First screening identified 3 candidate antigens. Of these, programmed cell death 11 (PDCD11) was determined to be associated with stroke (*p* < 0.0001), as evidenced from the second screening using AlphaLISA. The validation cohort revealed significantly higher antibody levels against PDCD11 (PDCD11-Ab levels) in patients with TIA than in HDs. Multivariate logistic regression analysis indicated that the predictive value of PDCD11-Ab levels for TIA [Odds ratio (OR): 2.44, 95% confidence interval (CI): 1.33-4.57, *p* = 0.0039] was not inferior to other known risk factors for ischemic stroke, including age (OR: 4.97, 95% CI: 2.67–9.48, *p* < 0.0001); hypertension (OR: 3.21, 95% CI: 1.76–5.86, *p* = 0.0001); and diabetes (OR: 4.31, 95% CI: 1.74–11.2, *p* = 0.0015).

**Conclusion:**

Serum PDCD11-Ab level may serve as a potential biomarker for TIA.

## INTRODUCTION

Ischemic stroke is one of the major causes of mortality and morbidity worldwide, and one of the primary pathological processes responsible for stroke is atherosclerosis [1–3]. Atherosclerosis shares characteristics of chronic inflammatory disease, and various immune cells have been reported to play roles in atherogenesis [4–8]. Proposed antigenic proteins related to atherogenesis that may be recognized by the immune system are oxidized low density lipoprotein (oxLDL), phosphorylcholine, heat shock proteins (Hsps), apolipoprotein A-1, and phospholipids [9].

Serological identification of antigens by recombinant cDNA expression cloning (SEREX), a combination of molecular cloning using phage expression libraries with serological typing, is an established method for identifying antigenic proteins [10, 11]. SEREX has been used to identify more than 1000 novel cancer antigens and is considered one of the most effective methods for identifying antigenic targets on a genomic scale [12–14]. Although this method was originally developed to screen cancer-associated antigens, it has been applied for vascular disorders such as transplant-associated coronary disease [15], Kawasaki disease [16], and moyamoya disease [17]. Previously, we have conducted SEREX screening for atherosclerotic diseases such as carotid artery stenosis [18] and cerebral infarction [19, 20] and identified RPA2, TUBB2C, ATP2B4, and BMP-1 as the associated antigens.

Recent clinical studies have focused on early intervention benefits in patients with transient ischemic attack (TIA) to prevent the subsequent development of cerebral infarction [21]. However, the diagnosis of TIA is sometimes difficult because of the lack of objective evidences detected by various medical examinations such as magnetic resonance imaging (MRI), echo cardiogram, and Holter electrocardiogram. In this context, the diagnosis of TIA by convenient blood analysis can be useful. In this study, we aimed to identify autoantibodies responsible for TIA by screening a human aortic endothelial cell cDNA library using the sera of patients with TIA.

## RESULTS

### First screening by expression cloning

**Figure 1:**
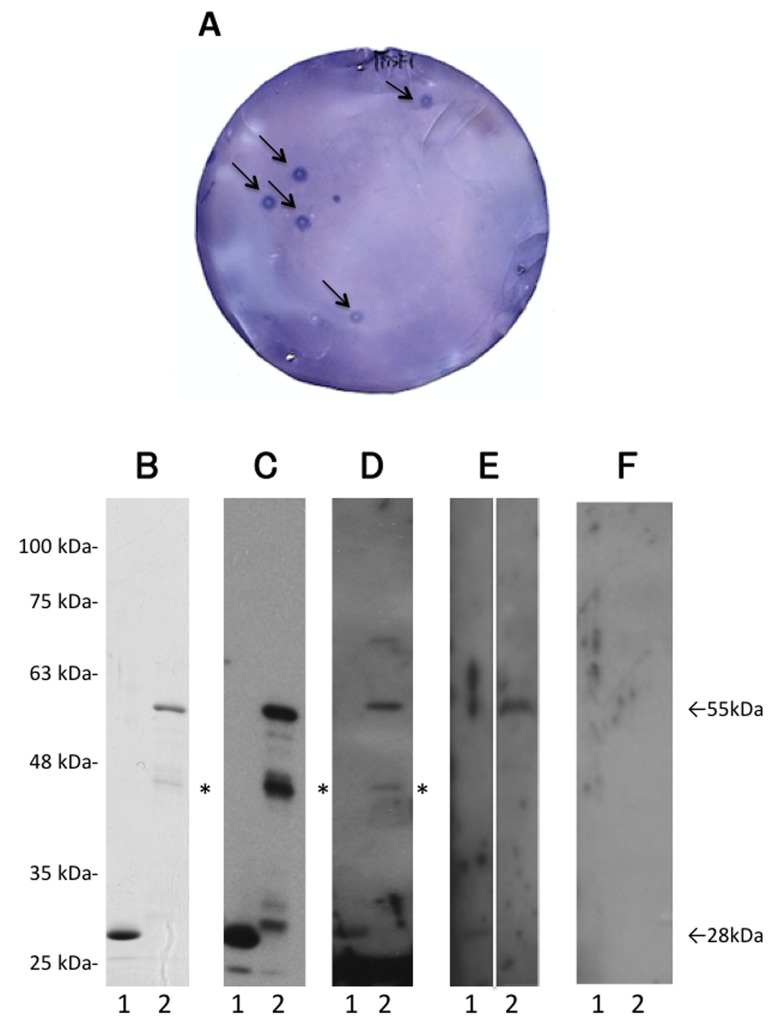
First screening by SEREX and Western blot analysis Recombinant proteins were blotted onto nitrocellulose membranes and reacted with patient serum. Arrows indicate positive phage clones. Positive clones were re-cloned for 2 additional times to obtain monoclonality **A.** GST protein (lane 1) and affinity-purified GST-tagged PDCD11 (lane 2) were separated on 11% SDS-polyacrylamide gels and stained with Coomassie Blue **B.**, or western blotted using anti-GST antibody **C.,** or the autologous sera of patients with aCI **D.**, TIA **E.**, and HD **F.** Asterisks indicate partially degraded proteins. Molecular weights are shown in the left.

We screened 2 × 10^6^ cDNA clones using sera of 20 patients with TIA and isolated 36 reacting clones (Figure 1A). DNA sequence analysis and a search for homologous sequences in an NCBI-accessible database indicated that these isolated clones comprised 18 independent genes. GST-fusion recombinant proteins were successfully produced using pGEX-4T vectors in 3 of 18 antigens (Table 1), which included programmed cell death 11 (PDCD11), catenin alpha 1 (CTNNA1), and ARP3 actin-related protein 3 homolog B (ACTR3B). The region of PDCD11 between amino acids 1583 and 1831 was obtained as a pBluescript II clone and recombined into pGEX 4T-3 expression vector. Similarly, the cloned regions of CTNNA1 and ACTR3B, which were amino acids 313-560 and 94-316, respectively, were recombined into pGEX 4T-3 and 4T-1 vectors, respectively.

**Table 1:** Genes of candidate antigen in 2nd screening

Gene name	Full name (Homology)	Accession No.	CDS	Site of cloned region
*PDCD11*	*programmed cell death 11 (PDCD11)*	NM_014976.1	88..5703	4833..5581
*CTNNA1*	*catenin alpha 1 (CTNNA1), transcript variant 1*	NM_001903	128..2848	1808..3801
*ACTR3B*	*ARP3 actin-related protein 3 homolog B (yeast) (ACTR3B), transcript variant 1*	NM_020445	135..1391	413..1084

### Secondary screening by AlphaLISA for antibodies against candidate antigens

**Figure 2:**
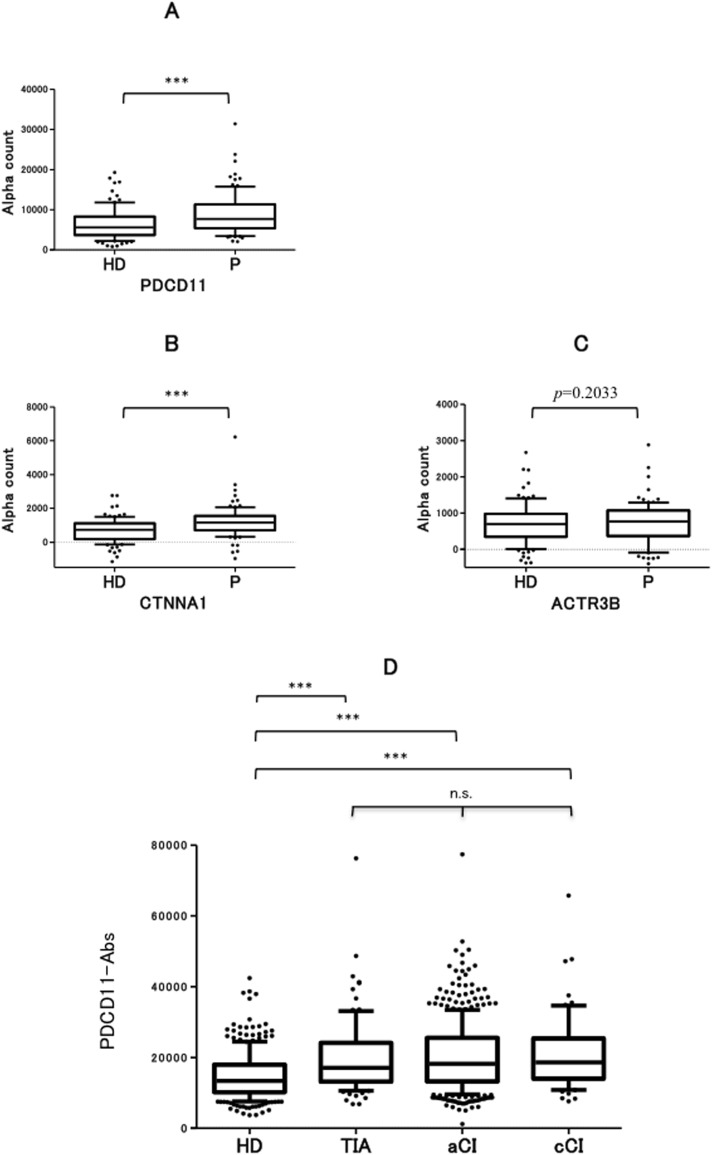
Serum antibody levels against SEREX antigens examined by AlphaLISA Antibody levels against 3 antigen candidates, PDCD11 **A.**, CTNNA1 **B.**, and ACTR3B **C.**, were compared between HDs and patients with aCI in second screening cohort. Alpha counts represent relative antibody levels. ***p < 0.0001 and *p* = 0.2033 were calculated by Mann–Whitney U test. **D.** The levels of PDCD11-Abs examined by AlphaLISA in the validation cohort. The PDCD11-Ab levels were compared between HDs and patients with TIA, aCI, or cCI. ***p < 0.001 was calculated by Mann–Whitney U test with type I error adjustment using Bonferroni procedure and not significant (n.s.), *p* = 1 was calculated by Kruskal–Wallis test with type I error adjustment using Bonferroni procedure. HD, healthy donors; P, patients with acute cerebral infarction; TIA, transient ischemic attack; aCI, acute cerebral infarction; cCI, chronic cerebral infarction; Ab, antibody.

In order to determine whether the 3 antigen candidates were related to ischemic stroke, we examined the antibody levels in patients with acute cerebral infarction (aCI) and healthy donors (HDs) in the second screening cohort. Serum antibody levels against PDCD11 (PDCD11-Ab levels) were significantly higher in patients with aCI than in HDs (*p* < 0.0001) (Figure 2A). Mean ± SD values of patients and HDs were 8843 ± 5217 and 6398 ± 3896, respectively. Antibody levels against CTNNA1 and ACTR3B were much lower as compared with those against PDCD11. A significant difference in CTNNA1 antibody levels was observed between HDs and patients, whereas no difference was noted in ACTR3B antibody levels (Figure 2B and 2C).

### Western blots of purified antigens

We confirmed the presence of PDCD11-Ab in patients’ sera by western blot analysis. GST-PDCD11 as well as GST proteins were recognized by the anti-GST antibody as 55-kDa and 28-kDa proteins, respectively (Figure 1B and 1C). The molecular weight of the largest product was similar to that predicted by sequencing analysis. Moreover, GST-PDCD11 reacted with serum antibodies of patients with aCI and TIA, but not with HD (Figure 1D-1F).

### Validation of elevated PDCD11-Ab levels in stroke patients

To validate the elevated levels of PDCD11-Abs in stroke patients, we further examined PDCD11-Ab levels in the independent validation cohort (n = 906). AlphaLISA revealed significantly higher PDCD11-Ab levels in patients with chronic cerebral infarction (cCI) (*p* < 0.0001), aCI (*p* < 0.0001), or TIA (*p* < 0.0001) as compared with HDs (Figure 2D). Mean ± SD values of patients with cCI, aCI, or TIA and HDs were 21230 ± 10413, 20138 ± 9617, 20126 ± 10491, and 14727 ± 6658, respectively. However, no significant differences in PDCD11-Ab levels were observed among patients with cCI, aCI, and TIA. Thus, PDCD11-Ab levels may be closely related to an ischemic brain lesion.

### Association between PDCD11-Ab levels and other clinical parameters in validation cohort

We then examined correlations between PDCD11-Ab levels and other clinical parameters in the validation cohort. A weak association was observed between PDCD11-Ab levels and age (r = 0.3082, *p* < 0.0001). PDCD11-Ab levels were higher in females than in males (*p* = 0.0073); moreover, the association was higher in patients with cardiovascular disease (CVD) than those without the disease (*p* = 0.0011). A strong association was observed between PDCD11-Ab levels and hypertension (*p* < 0.0001), diabetes (*p* = 0.0003), and stroke (*p* < 0.0001) (Supplementary Figure 1). No significant correlations were observed between PDCD11-Ab levels and other parameters, including hyperlipidemia, obesity, and smoking.

### Association between TIA and clinical parameters including PDCD11-Ab levels

The cutoff value of PDCD11-Ab for predicting of TIA was determined to be 13921 by ROC curve analysis with a sensitivity of 73.6% and a specificity of 55.8%. The area under the curve was 0.679. We used HDs and patients with TIA in the validation cohort to determine the cutoff value, which was validated in the validation cohort. Of 92 patients with TIA and 285 HDs, 66 patients with TIA and 126 HDs tested positive *(p* < 0.0001 was calculated using the chi-square test).

Results of univariate and multivariate logistic regression analyses are shown in Table 2. Using the cutoff value of 13921, univariate logistic regression analysis revealed that the elevated PDCD11-Ab level was associated with the increased risk of TIA (OR: 3.52, 95% CI: 2.09-5.93, *p* < 0.0001). Factors with a univariate *p* value of less than 0.05 were included in the multivariate analysis. Multivariate logistic regression analysis revealed that the elevated PDCD11-Ab level was an independent predictor of TIA (OR: 2.44, 95% CI: 1.33-4.57, *p* = 0.0039). Predictive value of PDCD11-Ab for TIA was not inferior to other known risk factors of TIA including age (OR: 4.97, 95% CI: 2.67-9.48, *p* < 0.0001); hypertension (OR: 3.21, 95% CI: 1.76-5.86, *p* = 0.0001); and diabetes (OR: 4.31, 95% CI: 1.74-11.2, *p* = 0.0015).

**Table 2:** Logistic regression of predictive factors for TIA (*n* = 377; no. of events = 92)

	Univariate analysis			Multivariate analysis		
	OR	95%CI	*p* value	OR	95%CI	*p* value
Age ( ≥ 60)	9.83	5.67-17.8	<0.0001	4.97	2.67-9.48	<0.0001
Male	0.73	0.45-1.18	0.2034			
HT	7.38	4.39-12.4	<0.0001	3.21	1.76-5.86	0.0001
DM	9.96	4.68-21.2	<0.0001	4.31	1.74-11.2	0.0015
HL	4.01	2.34-6.86	<0.0001	1.93	0.99-3.72	0.0509
CVD	8.23	1.57-43.2	0.0127	1.33	0.22-10.8	0.7610
Obesity (BMI ≥ 25)	1.09	0.66-1.82	0.7283			
Smoking	0.99	0.61-1.58	0.9569			
PDCD11 (> 13921)*	3.52	2.09-5.93	<0.0001	2.44	1.33-4.57	0.0039

* PDCD11, elevated PDCD11-Ab levels. PDCD11-Ab cutoff was 13921 based on ROC curve analysis.

HT, hypertension; DM, diabetes mellitus; HL, hyperlipidemia; CVD, cardiovascular disease; OR, odds ratio.

### Association of PDCD11-Ab levels with AMI and DM

We further examined correlations between PDCD11-Ab levels and acute myocardial infarction (AMI) and diabetes mellitus (DM) in the other validation cohort using sera of each 128 age-matched patients and HDs. AlphaLISA revealed no significant differences in PDCD11-Ab levels between patients with AMI and DM and HDs (Supplementary Figure 2).

### Expression of PDCD11 in human ischemic brain

**Figure 3:**
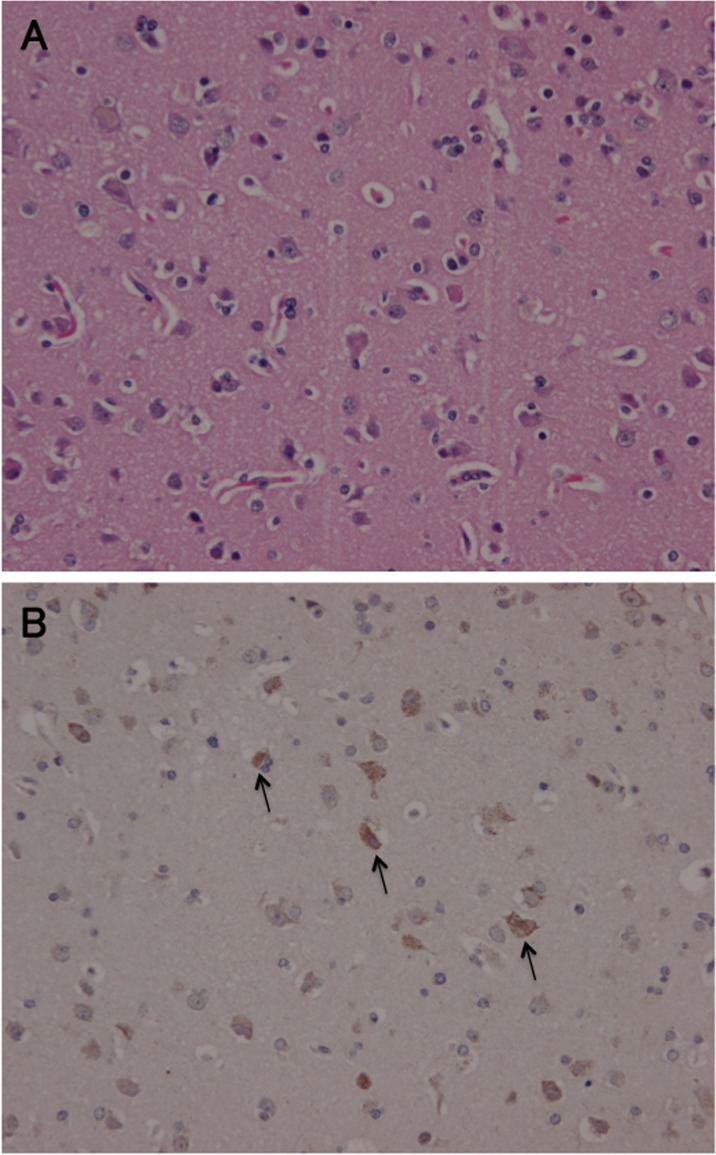
Immunohistochemistry Surgically-resected ischemic brain tissue was stained with hematoxylin only **A.**, and anti-PDCD11 antibody (**B.**, arrows).

Because PDCD11-Ab levels were elevated specifically in sera from patients with ischemic stroke, we further examined PDCD11 protein expression in surgically-resected ischemic brain tissue using immunohistochemistry. HE staining showed both viable and hollowing cells by necrosis co-existed in the ischemic penumbra (Figure 3A). Immunostaining with anti-PDCD11 demonstrated marked expression of PDCD11 in necrotic cells but not in normal cells (Figure 3B).

## DISCUSSION

The major finding of this study is that PDCD11-Ab levels were elevated in patients with symptomatic stroke, which turned out to be an independent predictor of TIA. In accordance with our knowledge, this is the first study that has identified autoantibodies against PDCD11, which were elevated in sera of TIA patients.

### Proposed mechanisms of elevation in PDCD11-Ab levels in patients with TIA and stroke

PDCD11 is a NF-κB-binding protein that colocalizes with U3 RNA in the nucleolus and is required for rRNA maturation and generation of 18S rRNA. PDCD11 is necessary for Fas ligand (FasL) expression, and PDCD11 overexpression is known to induce transcription of FasL (TNFSF6; 134638), leading to the induction of apoptosis through Fas/FasL/caspase death pathway [22–25]. It is also reported that post-stroke inflammatory response of FasL is an important contributing mechanism in ischemic brain lesion [26]. Therefore, it can be speculated that Fas/FasL/caspase death pathway may be activated in the ischemic brain tissue and that PDCD11 may be overexpressed. As the overexpression of cancer -related antigens stimulate the immunity and produce their autoantibodies, overexpression of PDCD11 in ischemic brain tissue might also stimulate autoimmune response and produce the PDCD11 autoantibody in patients with ischemic stroke. We further examined PDCD11 protein expression in surgically-resected ischemic brain tissue using immunohistochemistry to prove our speculation. PDCD11 expression was closely co-localized with ischemic brain cells (Figure 3).

We have screened autoantibody levels in the sera of patients with ischemic stroke and report TUBB2C [19], ATP2B4, BMP-1 [20], DHPS [27], and SH3BP5 [28] as possible antigens that may be implicated in the development of stroke. Antibody levels against these proteins were also elevated in the sera of patients with CVD and DM. Accordingly, we speculated that because ischemic stroke is only one of the phenotypes of whole body atherosclerosis, stroke associated autoantibodies we have detected may have also been elevated in the sera of patients with other atherosclerotic diseases. On the other hand, serum PDCD11-Ab levels were elevated only in patients with TIA and stroke but not in those with AMI and DM. This intriguing finding led us to hypothesize that the immune response against PDCD11 is not due to the leakage of PDCD11 from atherosclerotic plaques but due to the leakage from brain tissues exposed to ischemia. Leakage of the overexpressed PDCD11 from ischemic brain tissues may have induced autoimmune responses against PDCD11. Immunological research would further verify the mechanisms of PDCD11-Ab elevation in the sera of patients with TIA and stroke.

### Clinical implications of measuring PDCD11-Ab levels in TIA patients

Results of this study suggest that the measurement of serum PDCD11-Ab levels can provide valuable information for diagnosing TIA. According to a nationwide survey of patients with stroke, approximately 15% of patients with ischemic stroke experiences TIA before the onset of stroke [29]. Early medical intervention to TIA is known to prevent the development of subsequent stroke; therefore, early and accurate diagnosis of TIA is clinically significant. However, approximately half of patients with TIA visited medical facilities after their symptoms completely disappeared [30, 31], and the diagnosis of TIA is sometimes difficult because physicians have to diagnose TIA only by taking history into consideration. Clinical examinations such as MRI, echography of cardia and carotid artery, and Holter electrocardiogram may increase the diagnostic accuracy of TIA; however, these are expensive, time consuming, and inconvenient. Therefore, if we are able to diagnose TIA simply by blood examination, this will significantly contribute to the clinical practice in terms of preventing stroke development as well as facilitating medical economy.

Although the PDCD11-Ab was significantly elevated in sera of TIA patients independent of other cardiovascular risk factors, the diagnostic value of PDCD11 alone was weak (sensitivity and specificity were 73.6% and 55.8%, respectively). In our opinion, the diagnostic value will improve by a combination of the measurement of the PDCD11-Ab and clinical risk factors, including age, hypertension, and diabetes, which were independent predictive factors for TIA in the multivariate logistic regression analysis (Table 2). In fact, the positive predictive values with the combination of PDCD11-Ab and clinical risk factors were higher than those with clinical risk factors alone (Table 3). In addition, we are now trying to produce the peptide antigen with a localized epitope, which may improve the sensitivity and specificity.

**Table 3:** Validation of predictive factors for TIA (*n* = 377; no. of events = 92)

	Clinical risk factor			Clinical risk factor + PDCD11 (>13921)*		
	TIA(+)	TIA(−)	PPV	TIA(+)	TIA(−)	PPV
Age ( ≥ 60)	72	79	47.7%	55	40	57.9%
HT	59	57	50.9%	49	30	62.0%
DM	26	11	70.3%	22	3	88.0%
Age ( ≥ 60) + HT	52	28	65.0%	43	15	74.1%
Age ( ≥ 60) + DM	24	5	82.8%	21	1	95.5%
HT + DM	20	5	80.0%	18	1	94.7%
Age ( ≥ 60) + HT + DM	20	2	90.9%	18	0	100%

* PDCD11, elevated PDCD11-Ab levels. PDCD11-Ab cutoff was 13921 based on ROC curve analysis.

HT, hypertension; DM, diabetes mellitus; PPV, positive predictive value.

On the other hand, the multivariate logistic regression analysis revealed that the elevation of PDCD11-Ab levels was a predictive marker for TIA. Thus, we planned to examine PDCD11-Ab by a blood test during the regular medical check-up. If HDs without any symptoms have a high level of PDCD11-Ab on this test, they could have high risks for TIA or stroke. Hence, examinations should be conducted more carefully.

## CONCLUSION

In this study, serum PDCD11-Ab levels were found elevated in patients with TIA when compared with HDs; thus, it can serve as a biomarker for TIA. Moreover, patients with elevated PDCD11-Ab levels may require a more careful and intensive treatment.

## MATERIALS AND METHODS

### Ethical approval

This study was approved by the Local Ethical Review Board of the Graduate School of Medicine, Chiba University, as well as those of co-operating hospitals, and it was performed in accordance with the principles of the Declaration of Helsinki. Recombinant DNA studies were performed with official permission from the Graduate School of Medicine, Chiba University, and conducted in conformity with the rules of the Japanese government. Written informed consents were obtained from all participants.

### Sera from patients and healthy donors

Patients who suffered ischemic stroke and were admitted to 3 participant hospitals within 2 weeks from stroke onset were included in the study. Healthy donors (HDs) included individuals without a history of ischemic stroke who underwent medical checkups including cerebral MRI. Patients with autoimmune diseases were excluded from this study. The study population consisted of 3 independent cohorts: first screening, second screening, and validation cohort.

Twenty Japanese adults with TIA were selected for the first screening cohort, and the second screening involved 96 patients with acute cerebral infarction (aCI) and 96 HDs. In order to evaluate relations between antibody levels and other clinical parameters associated with ischemic stroke, 621 patients and 285 HDs were allocated to the validation cohort. Of 621 patients in the validation cohort, 65, 464, and 92 suffered chronic cerebral infarction (cCI), aCI, and TIA. Clinical characteristics of patients and HDs are shown in Table 4. Furthermore, to validate the relations of antibody levels with acute myocardial infarction (AMI) and diabetes mellitus (DM), each 128 age-matched patients and HDs were selected.

**Table 4:** Baseline characteristics of subjects

	1st	2nd Screening		Validation			
	TIA (*n* = 20)	aCI (*n* = 96)	HD (*n* = 96)	Stroke (*n* = 621)			HD (*n* = 285)
				cCI (*n* = 65)	aCI (*n* = 464)	TIA (*n* = 92)	
Age (years)	67.5 ±19.1	67.7** ±12.7	56.3 ±7.1	73.3** ±9.2	75.5** ±11.5	70.2** ±11.6	52.3 ±11.7
Male gender	12 (60.0%)	73* (76.0%)	53 (55.2%)	48 (73.8%)	271 (58.4%)	55 (59.7%)	188 (65.9%)
Hypertension	13 (65.0%)	61** (63.5%)	21 (21.9%)	53** (81.5%)	335** (72.2%)	60** (65.2%)	57 (20.0%)
Diabetes	7 (35.0%)	30** (31.3%)	5 (5.2%)	22** (33.8%)	125** (26.9%)	26** (28.3%)	11 (3.9%)
Hyperlipidemia	8 (40.0%)	40** (41.7%)	10 (10.4%)	25** (38.5%)	122** (26.3%)	36** (39.1%)	40 (14.0%)
CVD	1 (5.0%)	7 (7.3%)	3 (3.1%)	2** (3.1%)	40** (8.6%)	5** (5.4%)	0 (0.0%)
Obesity (BMI ≥ 25)	5 (25.0%)	24** (25.0%)	31 (32.3%)	11 (16.9%)	127 (27.4%)	30 (32.6%)	88 (30.9%)
Smoking	11 (55.0%)	68** (70.8%)	21 (21.9%)	33 (50.8%)	228 (49.1%)	43 (46.7%)	132 (46.3%)

Data represents means (±SD) for continuous data and *n* (%) for categorical data.

*p < 0.01 versus HD, **p < 0.001 versus HD.

TIA, transient ischemic attack; aCI, acute cerebral infarction; HD, healthy donor; cCI, chronic cerebral infarction; CVD, cardiovascular disease.

Sera of patients with TIA, cCI, and aCI were obtained from Chiba Prefectural Sawara Hospital, Chiba Rosai Hospital, and Chiba Aoba Municipal Hospital. Sera of patients with AMI and DM were obtained from Kyoto University Hospital and Chiba University Hospital, respectively. Sera of HDs were obtained from Chiba Prefectural Sawara Hospital, Higashi Funabashi Hospital, and Port Square Kashiwado Clinic. After collection, samples were centrifuged at 3,000 ×g for 10 min at room temperature, and the supernatants were stored at −80°C until use. Repeated thawing and freezing of samples were avoided.

### Clinical data

Data regarding the risk factors for atherosclerosis, including age, gender, hypertension, diabetes, hyperlipidemia, cardiovascular disease (CVD), obesity, and smoking were collected from patients’ clinical records. Hypertension was defined as a history of systolic blood pressure > 140 mmHg, diastolic blood pressure > 90 mmHg, or use of antihypertensive agents. Diabetes was defined as having a history of diabetes diagnosed and/or treated with medication and/or fasting blood glucose ≥ 126 mg/dl. Hyperlipidemia was defined as a history of total cholesterol > 220 mg/dL, triglycerides > 150 mg/dL, or use of lipid-lowering agents. CVD was defined the presence of myocardial infarction or angina pectoris histories. Patients were considered smokers if they smoked during the study period or had a history of smoking, and obesity was defined as body mass index (BMI) ≥ 25. The definition of TIA was determined by the presence of a transient episode of neurological dysfunction caused by focal brain, spinal cord, or retinal ischemia, without acute infarction [32].

### Screening by expression cloning

Immunoscreening was performed using a modified version of previously published methods [18–20, 33, 34]. We used a commercially available human aortic endothelial cell cDNA library (Uni-ZAP XR Premade Library, Stratagene, La Jolla, CA) to screen for clones that were immunoreactive against sera of patients with TIA. *Escherichia coli* (*E. coli*) XL1-Blue MRF′ was infected with Uni-ZAP XR phage, and the expression of resident cDNA clones was induced after blotting inflectional bacteria onto nitrocellulose membranes (NitroBind, Osmonics, Minnetonka, MN), which were pretreated with 10 mM isopropyl-β-D-thiogalactoside (IPTG) (Wako Pure Cemicals, Osaka, Japan) for 30 min. Membranes with bacterial proteins were washed 3 times with TBS-T [20 mM Tris-HCl (pH 7.5), 0.15 M NaCl and 0.05% Tween-20], and nonspecific binding was blocked by incubating membranes with 1% protease-free bovine serum albumin (Nacalai Tesque, Inc., Kyoto, Japan) in TBS-T for 1 h. Membranes were then incubated overnight with 1:2000 diluted sera of patients. After 3 washes with TBS-T, membranes were incubated for 1 h with 1:5000 diluted alkaline phosphatase-conjugated goat anti-human IgG (Jackson ImmunReseach Laboratories, West Grove, PA). Positive reactions were visualized by incubating membranes in a color development solution [100 mM Tris-HCl (pH 9.5), 100 mM NaCl, 5 mM MgCl_2_] containing 0.15 mg/ml of 5-bromo-4-chloro-3-indolylphospate (Wako Pure Chemicals) and 0.3 mg/ml of nitro blue tetrazolium (Wako Pure Chemicals). Positive clones were re-cloned for 2 additional times in order to obtain monoclonality as previously described [18–20, 33, 34].

### Sequence analysis of identified antigens

Monoclonalized phage cDNA clones were converted to pBluescript phagemids by *in vivo* excision using ExAssist helper phage (Stratagene). Plasmid DNA was obtained from the *E. coli* SOLR strain after transformation with the phagemid. Inserted cDNAs were sequenced and analyzed for homology with a public database provided by the National Center for Biotechnology Information (NCBI) (http://www.ncbi.nlm.nih.gov/Blast.cgi/).

### Construction of expression vectors

The expression plasmids of glutathione-S-transferase (GST)-fused proteins were constructed by recombining the cDNA sequences into pGEX-4T vectors (GE Healthcare Life Sciences, Pittsburgh, PA), as previously described [18–20, 34]. The pBluescript plasmids containing cDNA inserts were digested with *EcoRI* and *XhoI* and separated via agarose gel electrophoresis. Inserted cDNA fragments were isolated using GenElute^TM^ Minus EtBr Spin Columns (Merck, Darmstadt, Germany) and were ligated in frame to pGEX-4T using a Ligation-Convenience Kit (Nippon Gene, Toyama, Japan). Ligation mixtures were used to transform ECOS^TM^-competent *E. coli* BL-21 (Nippon Gene).

### Purification of recombinant candidate protein

Transformed *E. coli* BL-21 cells containing pGEX-4T clones were cultured in 200 ml of Luria broth and treated with 0.1 mM IPTG for 3 h. IPTG-treated cells were then harvested, washed with phosphate-buffered saline (PBS), and lysed by sonication in BugBuster Master Mix (Merck). Subsequently, cell lysates were centrifuged at 13,000 × g for 10 min at 4°C. Precipitates containing recombinant proteins were dissolved in 8 M urea in TED buffer [50 mM Tris-HCl (pH 8.0), 1 mM EDTA, and 1 mM dithiothreitol], followed by stepwise dialysis using 4 M and 2 M urea in TED buffer for 1 h each. Samples were further dialyzed using TED buffer for more than 12 h and were centrifuged at 10,000 × g for 30 min at 4°C. Recombinant proteins recovered in the supernatant were purified using glutathione-Sepharose column chromatography (GE Healthcare Life Sciences) according to the manufacturer’s instructions, and the purified proteins were concentrated using Amicon Ultra-15 Centrifugal Filter Device (Merck) [35].

### Western blotting

GST and GST-PDCD11 proteins (0.4 μg) were electrophoresed on SDS-polyacrylamide gels followed by western blotting using anti-GST antibody (Rockland, Gilbertsville, PA) or sera from patients with aCI or TIA. After incubation with horseradish peroxidase-conjugated secondary antibody, immunoreactivity was detected with Immobilon (Merck), as previously described [18, 36, 37].

### AlphaLISA

Amplified luminescent proximity homogeneous assay-linked immunosorbent assay (AlphaLISA) was performed using 384-well microtiter plates (white opaque OptiPlate^TM^, Perkin Elmer, Waltham, MA) containing 2.5 μl of 1:100 diluted sera and 2.5 μl of GST or GST-fusion proteins (10 μg/ml) in AlphaLISA buffer (25 mM HEPES (pH 7.4), 0.1% casein, 0.5% Triton X-100, 1 mg/ml dextran-500, and 0.05% Proclin-300). The reaction mixture was incubated at room temperature for 6-8 h. Next, anti-human IgG-conjugated acceptor beads (2.5 μl of 40 μg/ml) and glutathione-conjugated donor beads (2.5 μl of 40 μg/ml) were added and incubated further for 7 days at room temperature in the dark. The chemical emission was examind using EnSpire Alpha microplate reader (Perkin Elmer). Specific reactions were calculated by subtracting Alpha values of GST control from the values of GST-fusion proteins.

### Immnohistochemistry

Tissue samples were obtained from surgically resected brain of a patient with cerebral infarction. The samples were fixed with formalin and embedded in paraffin. The samples were pretreated by heating them in a heating citrate buffer at 98°C for 40 min. The first antibodies used were the monoclonal anti-human PDCD11 antibody (Atras Antibodies, Stockholm, Sweden) at a diluteion of 1:100 and incubated overnight at 4°C. The specimens were incubated with biotin-labeled rabbit anti-mouse/rabbit-IgG secondary antibody and, next, with streptavidin-labeled peroxidase. Section were counter-stained with hematoxylin after the DAB reaction as described in the literature [12].

### Statistical analyses

For continuous variables, Mann-Whitney U test or Kruskal-Wallis test were performed as appropriate. For categorical variables, chi-square test was performed. Univariate and multivariate logistic regression analyses were performed to identify the set of variables that could classify participants according to the presence of a history of ischemic stroke. The cutoff value of PDCD11-Ab levels for predicting stroke was determined to maximize the sum of sensitivity and specificity by receiver operating characteristic (ROC) curve analysis. All comparisons were planned and the tests were two-tailed. A *p* value of less than 0.05 was considered statistically significant. Univariate and multivariate logistic regression analyses and ROC curve analyses were performed using JMP Pro 13.0.0 software (SAS Institute Inc., NC), and other analyses were done using GraphPad Prism 5 (GraphPad Software, La Jolla, CA).

## SUPPLEMENTARY MATERIALS FIGURES


