# Supplementary material for: Elevation of autoantibody level against PDCD11 in patients with transient ischemic attack
Source: Oncotarget. 2017 Dec 24;9(10):8836–48. doi: 10.18632/oncotarget.23653 (PMC5823671; doi:10.18632/oncotarget.23653)
Supplement: Supplementary file 1 [file oncotarget-09-8836-s001.pdf]

Elevation of autoantibody level against PDCD11 in patients with transient ischemic attack

SUPPLEMENTARY MATERIALS

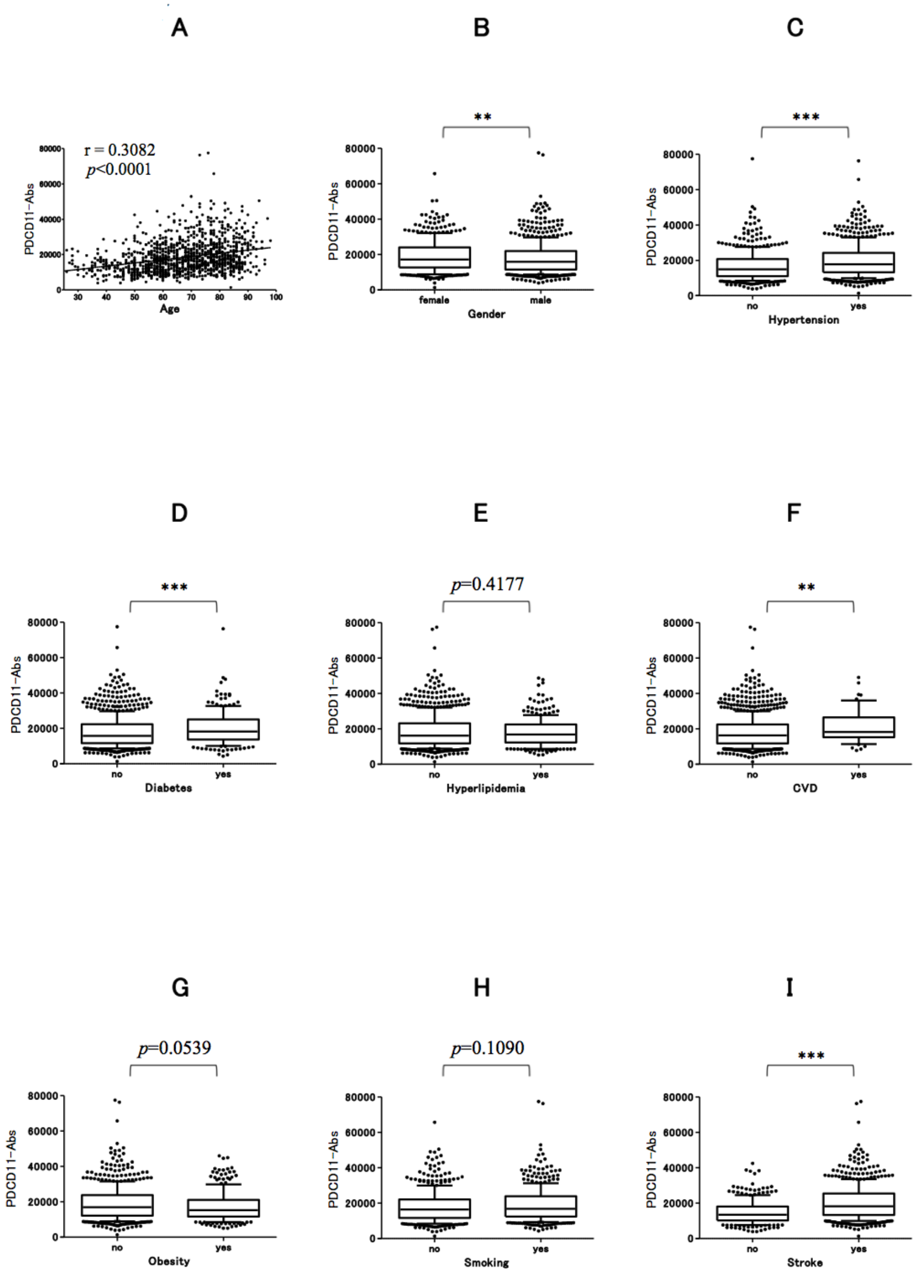

Supplementary Figure 1: Association between PDCD11-Ab levels and other clinical parameters in stroke patients. Correlations between PDCD11-Ab levels and age (**A**), gender (**B**), hypertension (**C**), diabetes (**D**), hyperlipidemia (**E**), CVD (**F**), obesity (BMI ≥ 25) (**G**), smoking habit (**H**), and ischemic stroke (**I**). Spearman's correlation analysis (**A**) and Mann–Whitney U test (B-I) were used. $^{**}p < 0.01$, $^{***}p < 0.001$. PDCD11-Ab, PDCD11 antibodies; CVD, cardiovascular disease.

A

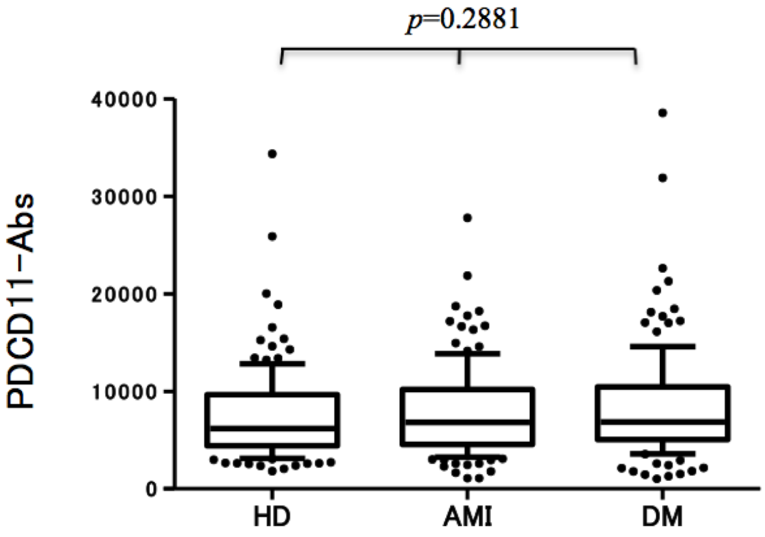

Supplementary Figure 2: Association between PDCD11-Ab levels and other atherosclerotic diseases including AMI and DM (A). The mean age ± SDs of HDs and patients with AMI or DM were 58.29 ± 5.63, 58.28 ± 8.5, and 58.37 ± 9.11, respectively. The serum samples of patients with AMI were obtained from Kyoto University Hospital, and those of patients with DM were obtained from Chiba University Hospital. Kruskal–Wallis test was used to calculate p values. AMI, acute myocardial infarction; DM, diabetes mellitus; HD, healthy donors.
